# Plasma Levels of Aβ42 and Tau Identified Probable Alzheimer’s Dementia: Findings in Two Cohorts

**DOI:** 10.3389/fnagi.2017.00226

**Published:** 2017-07-24

**Authors:** Lih-Fen Lue, Marwan N. Sabbagh, Ming-Jang Chiu, Naomi Jing, Noelle L. Snyder, Christopher Schmitz, Andre Guerra, Christine M. Belden, Ta-Fu Chen, Che-Chuan Yang, Shieh-Yueh Yang, Douglas G. Walker, Kewei Chen, Eric M. Reiman

**Affiliations:** ^1^Laboratory of Neuroregeneration, Banner Sun Health Research Institute, Sun City AZ, United States; ^2^Arizona State University-Banner Neurodegenerative Disease Research Center, Biodesign Institute, Arizona State University, Tempe AZ, United States; ^3^Cleo Roberts Center for Clinical Research, Banner Sun Health Research Institute, Sun City AZ, United States; ^4^Department of Neurology, National Taiwan University Hospital, College of Medicine, National Taiwan University Taipei, Taiwan; ^5^Graduate Institute of Brain and Mind Sciences, College of Medicine, National Taiwan University Taipei, Taiwan; ^6^Department of Psychology, National Taiwan University Taipei, Taiwan; ^7^Department of Statistics, College of Letters and Sciences, University of California, Berkeley, Berkeley CA, United States; ^8^Banner Alzheimer’s Institute, Phoenix AZ, United States; ^9^Department of Medical Imaging, National Taiwan University Hospital, College of Medicine, National Taiwan University Taipei, Taiwan; ^10^MagQu Co. Ltd. New Taipei City, Taiwan; ^11^Translational Genomics Research Institute, Phoenix AZ, United States; ^12^Arizona Alzheimer’s Consortium, Phoenix AZ, United States

**Keywords:** plasma, amyloid β, tau, Alzheimer’s disease, immunomagnetic reduction assay

## Abstract

The utility of plasma amyloid beta (Aβ) and tau levels for the clinical diagnosis of Alzheimer’s disease (AD) dementia has been controversial. The main objective of this study was to compare Aβ42 and tau levels measured by the ultra-sensitive immunomagnetic reduction (IMR) assays in plasma samples collected at the Banner Sun Health Institute (BSHRI) (United States) with those from the National Taiwan University Hospital (NTUH) (Taiwan). Significant increase in tau levels were detected in AD subjects from both cohorts, while Aβ42 levels were increased only in the NTUH cohort. A regression model incorporating age showed that tau levels identified probable ADs with 81 and 96% accuracy in the BSHRI and NTUH cohorts, respectively, while computed products of Aβ42 and tau increased the accuracy to 84% in the BSHRI cohorts. Using 382.68 (pg/ml)^2^ as the cut-off value, the product achieved 92% accuracy in identifying AD in the combined cohorts. Overall findings support that plasma Aβ42 and tau assayed by IMR technology can be used to assist in the clinical diagnosis of AD.

## Introduction

Definitive diagnosis of Alzheimer’s disease (AD) is based on neuropathological criteria for the density of amyloid and neuritic plaques, primarily composed of amyloid beta (Aβ) peptides, and neurofibrillary tangles, primarily composed of aggregated and phosphorylated tau (ptau). The constituents of these pathological hallmarks have been intensely investigated as diagnostic biomarkers for early AD in living subjects. There has been increasing use of positron emission tomography (PET) to detect fibrillar Aβ and tau pathologies in living subjects, along with biochemical measurements of Aβ42, Aβ40, tau, and ptau in the cerebrospinal fluid (CSF), for research and clinical studies of AD (Galasko et al., 1998; Shaw et al., 2009; Trojanowski et al., 2010; Molinuevo et al., 2014; Beckett et al., 2015; Kang et al., 2015; Kleinschmidt et al., 2015; Weiner et al., 2015; Leuzy et al., 2016). However, clinical imaging is limited by high cost, while lumbar puncture for CSF collection is not a readily acceptable procedure for many elderly patients (Lowery and Oliver, 2008; Amorim et al., 2012; Zetterberg et al., 2013; Alcolea et al., 2014; Duits et al., 2015). In addition, there is still lack of consensus as to how CSF profiles of Aβ42, tau, and ptau should be used in clinical settings for disease diagnosis (Dumurgier et al., 2013; Blennow et al., 2015).

Blood is an inexpensive and convenient source for developing disease biomarkers. Well-validated blood biomarker panels could assist in diagnosis, patient screening for clinical trials, and follow-up studies. To date, the levels of Aβ, tau, and ptau measured in plasma or serum samples reported in the literature have been inconsistent [reviewed in (Hampel et al., 2010; Toledo et al., 2013)]. The concentrations of these markers in plasma or serum are 10- to 100-fold lower than in CSF, which has caused challenges for accurate and reliable measurement when conventional enzyme-linked immunosorbent assays (ELISA) have been used (Rissin et al., 2013; Blennow and Zetterberg, 2015b).

To meet the need of higher detection sensitivity, new technologies have been developed (Hye et al., 2006; Yang et al., 2011; Chang et al., 2012; Irwin et al., 2012; Mattsson et al., 2013). Among these technologies, immunomagnetic reduction (IMR) assays can sensitively quantify levels of Aβ and tau in biofluids by detecting the reduction in magnetic signals after binding of magnetic nanobead-conjugated antibody to the target analytes using a high-sensitivity magneto-susceptometer, a superconducting quantum interference device (SQUID) (Yang et al., 2011).

Studies in Taiwanese cohorts using this technology showed significant increases in Aβ42 and tau concentrations in plasma samples of subjects with mild cognitive impairment (MCIs) due to AD, and in early-stage AD (Chiu et al., 2012). A similar study also showed that the computed products of Aβ42 and tau increased the sensitivity for discriminating normal controls (NCs) from early AD subjects (ADs), and MCIs from early ADs (Chiu et al., 2013).

While previous IMR studies have provided promising findings, the assays have not been independently confirmed in other cohorts of different ethnicity. To achieve this, we recruited a cohort of NC and AD subjects from the Banner Sun Health Research Institute (BSHRI) (Sun City, AZ, United States) to provide plasma samples for IMR assays. We compared the plasma levels of Aβ42 and tau in the BSHRI cohort with those of samples collected independently at the National Taiwan University Hospital (NTUH). Using the same statistical approach, we determined the ability of these measures to distinguish ADs from NCs in both cohorts. To further explore the utility of plasma Aβ, Aβ42, and tau levels for AD diagnosis across age and population, we developed new statistical models that incorporated age along with the levels of these biochemical analytes to establish cut-off values for identifying AD in the combined data from the BSHRI and NTUH cohorts. The findings confirmed that plasma Aβ42 and tau levels detected by IMR technology achieved exceptional accuracy of diagnosis and thus have potential to be further developed as blood biomarkers for assisting in the clinical diagnosis of AD.

## Materials and Methods

### Participants

#### BSHRI Site (United States)

The participants consisted of 16 healthy non-demented control subjects (NCs) who were also enrolled in the Arizona Study of Aging and Neurodegenerative Disorders (ASAND), and 16 age-matched subjects clinically diagnosed as probable Alzheimer’s dementia (ADs) by a dementia neurologist. The recruitment period was from January to July, 2014.

All participants were 65 years or older, from both genders, and with a minimum secondary school education. Probable AD dementia subjects received this diagnosis according to the 2011 NIA-AA diagnostic guidelines with Functional Assessment Staging (FAST) scores of 4–6 and Mini Mental State Examination (MMSE) scores of 10–20. The inclusion criteria of NCs were subjects without depression syndrome, with FAST scores of 1–2 and MMSE scores of 28–30. Although NCs had been assessed with a battery of neuropsychological tests as a consequence of participation in ASAND, we only included MMSE in statistical analysis because neuropsychological assessments of non-ASAND participants had been limited to this measure. Subjects with medical history of major systemic diseases that could affect cognitive functions, including cardiopulmonary failure, hepatic or renal failure, diabetes, head injury, stroke or other neurodegenerative disease, were excluded.

#### NTUH Site (Taiwan)

This study consisted of 63 NCs and 31 ADs. The recruitment of the subjects was described previously (Chiu et al., 2012). NCs received physical and neurological examinations and were scored less than 9 on a short-form Geriatric Depression Scale (GDS-S) and confirmed as cognitively normal with a battery of neuropsychological tests. Subjects with AD were recruited from the memory clinic at the NTUH and fulfilled the NINCDS/ADRDA criteria for probable AD (McKhann et al., 1984).

### Approval of Human Subject Protocols

Both BSHRI and NTUH studies were conducted according to human subject study protocols approved, respectively, by the Institutional Review Boards of the two study sites (BSHRI: Western Institutional Review Board, www.wirb.com; NTUH: National Taiwan University Hospital Institutional Review Board). Subject enrollment and consenting processes were conducted by qualified personnel. Written informed consent was obtained from each participant or a qualified representative (spouse or family member).

### Blood Sample Processing

The plasma samples were collected for this study employing centrifugation speeds of 2,500 × *g*. This differed from the ADNI recommended protocol of 1,500 × *g*, but was used to ensure consistency with previous studies using IMR-based assays (Chiu et al., *2*013). Approximately 16 ml of whole blood was collected into EDTA-treated tubes. Samples were centrifuged at 2,500 × *g* for 15 min at room temperature within 15 min of the blood draw. Plasma was removed, aliquoted into various volumes, and stored at -80°C. White blood cell pellets were used for determination of apolipoprotein E (ApoE) genotype from each case recruited at BSHRI; the ApoE genotypes were not available from the subjects recruited at NTUH site. Frozen plasma aliquots were shipped on dry ice to MagQu Co. Ltd.^1^ (New Taipei City, Taiwan) for IMR assays. Assays were carried out without knowledge of individual identification or diagnosis.

### IMR Assays

The technical details of the IMR assays have been described previously (Yang et al., 2011; Chiu et al., 2013, 2014; Tzen et al., 2014). The selection of the antibodies conjugated to the IMR reagents (MagQu Co. Ltd.; Catalog Numbers: MF-AB2-0060 and MF-TAU-0060) were based on epitopes, affinity to antigens, ability to be conjugated onto MagQu magnetic nanobeads, and the ability to provide linearity of standard curves quantified by magnetic signal reduction. The company tested these reagents in human plasma samples spiked with the proteins used for making standards for measurement accuracy. The amounts of the proteins added were 95–1,038 pg/ml and 100–1,197 pg/ml respectively for Aβ42, and tau. The recovery rates of these spiked proteins, expressed in mean% (+standard error), were 98.98% (+1.63) for Aβ42 and 96.75% (+2.26) for tau. To assay tau, 40 μl of plasma sample was mixed with 80 μl IMR reagents at room temperature. To assay Aβ42, 60 μl of plasma sample was mixed with 60 μl of IMR reagent.

The analyzer for IMR assays is a SQUID-based alternate current (ac) magnetosusceptometer (model X*ac*Pro-S, MagQu Co., New Taipei City, Taiwan). It detects magnetic signal changes during the course of antigen and antibody interactions, expressed as percentage reductions of immunomagnetic signals (IMR%), which are then converted to sample concentrations using values from the standard curves of the respective analytes. The reduction of oscillation detected by SQUID corresponds to the amounts of analytes bound to the antibodies.

### Statistical Analysis

**FIGURE 1:**
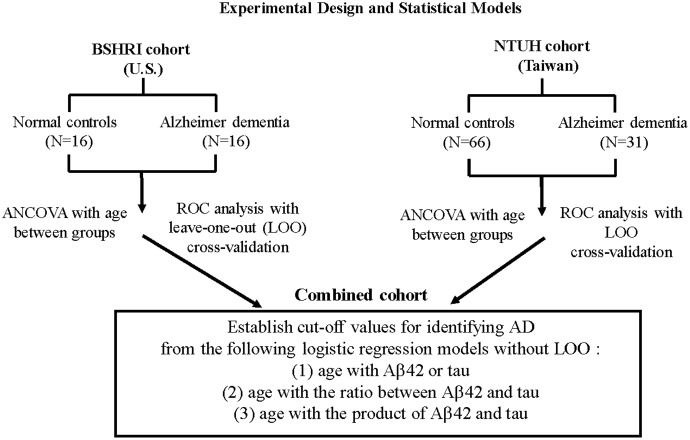
Experimental design and statistical models.

Basic hypothesis-testing analyses were performed using the statistical package SPSS (version 22, IBM). All ROC analyses and performance comparisons were completed using R^2^ (version 3.2.4). Two statistical objectives were set for both separate and combined cohorts (**Figure 1**). First, we tested the hypothesis that probable ADs are different from NCs in each of the analytes (Aβ42 and tau) and in the ratios and products of Aβ42 and tau. Age was included as a covariate in determinations of group differences using analysis of covariance (ANCOVA).

Second, to examine the corresponding criteria of distinguishing probable ADs from NCs to assist on clinical diagnosis, we used receiver operational characteristics (ROC) curve analysis. For adequate objective assessment, performance metrics based on the area under the curve (AUC) of ROC were compared using leave-one-out (LOO) cross-validation. Sensitivity, specificity, overall accuracy, positive likelihood ratio, negative likelihood ratio with their respective 95% confident interval (CI) except AUC were computed based on the logistic model. Age was included as an additional predictor together with the concentrations of single Aβ42 or tau analytes, or their combinations (ratios or products) in the logistic models.

## Results

### Participant Features

The demographic features for the BSHRI, NTUH, and combined cohorts are shown in **Table 1** according to disease group. The BSHRI participants were age- and gender-matched between NCs and ADs. However, the ADs in the NTUH cohort were significantly older than NCs (NCs: 64.4 + 1.1 years; ADs: 72.5 + 1.5 years, *P* = 0.003). Overall, the BSHRI cohort was significantly older in both groups (NCs: 81.9 + 1.5 years; ADs: 82.5 + 1.4 years). In the combined cohort, mean age was 75.9 + 1.4 years in ADs and 64.1 + 1.3 years in NCs (*P* = 0.0001). The MMSE scores of ADs compared to the Taiwanese cohort (BSHRI: 16.13 + 0.97; NTUH: 22.7 + 3.2) were significantly lower than those of NCs (BSHRI: 29.3 + 0.3; NTUH: 28.9 + 1.3; *P* < 0.001), indicating that ADs in BSHRI cohort were cognitively more impaired than the ADs in NTUH cohort. The MMSE scores of the combined cohort were 20.5 + 4.6 for ADs and 28.7 + 1.3 for NCs (*P* < 0.0001). As we compared the percentages of female subjects in each disease category, there were no significant differences between diagnostic groups within same cohort using Chi-squared analysis (NTUH: *P* = 0.1740; BSHRI: *P* = 0.2658). The ApoE genotype information was only available from the subjects recruited at the BSHRI site. Due to small number of subjects in BSHRI, we only determine the effect by the presence (carriers) or absence (non-carriers) of ApoE *𝜀*4. The results of one-way ANOVA analysis showed no statistical difference between non-carriers and carriers in ApoE *𝜀*4 in the levels of Aβ42 (means + standard errors: in NCs: non-carriers: 15.44 + 0.70 pg/ml, *N* = 10; carriers: 15.13 + 0.90 pg/ml, *N* = 6, *P* = 0.821; in ADs: non-carriers: 17.09 + 0.99 pg/ml, *N* = 5; carriers: 16.67 + 0.67 pg/ml, *N* = 11, *P* = 0.622) or tau (means + standard errors: in NCs: non-carriers: 19.22 + 3.61 pg/ml, *N* = 10; carriers: 22.59 + 4.66 pg/ml, *N* = 6, *P* = 0.196; in ADs: non-carriers: 37.31 + 5.10 pg/ml, *N* = 5; carriers: 33.25 + 3.44 pg/ml, *N* = 11, *P* = 0.633).

**Table 1:** Age, gender, and MMSE scores in the studied cohorts.

Cohort	BSHRI		NTUH		Combined	
Group	NC	AD	NC	AD	NC	AD
Subject No.	16	16	61	31	77	47
Male:Female	4:12	7:9	24:37	17:14	28:49	24:23
Apo E4 carrier:Non-carrier	6:10	11:5	–	–	–	–
Age, years	81.9 ± 1.5	82.5 ± 1.4	64.2 ± 1.1^a^	72.5 ± 1.8^a^	68.1 ± 1.3^b^	75.9 ± 1.4^b^
MMSE	29.3 ± 0.3^c^	16.1 ± 1.0^c^	28.9 ± 1.3^d^	22.7 ± 3.2^d^	28.7 ± 1.3^e^	20.5 ± 4.6^e^

*Abbreviations: BSHRI, Banner Sun health Research Institute; NTUH, National Taiwan University Hospital; NC, normal controls; AD, Alzheimer’s disease; MMSE, Mini Mental State Examination. Age and MMSE are shown in mean + standard errors. Lower case a to e: same letter denotes significant differences between the noted two values based on two-sample independent *t*-test (*P* < 0.05). –: not calculated*.

### AD-Associated Variations in Plasma Aβ42 and Tau Levels

**FIGURE 2:**
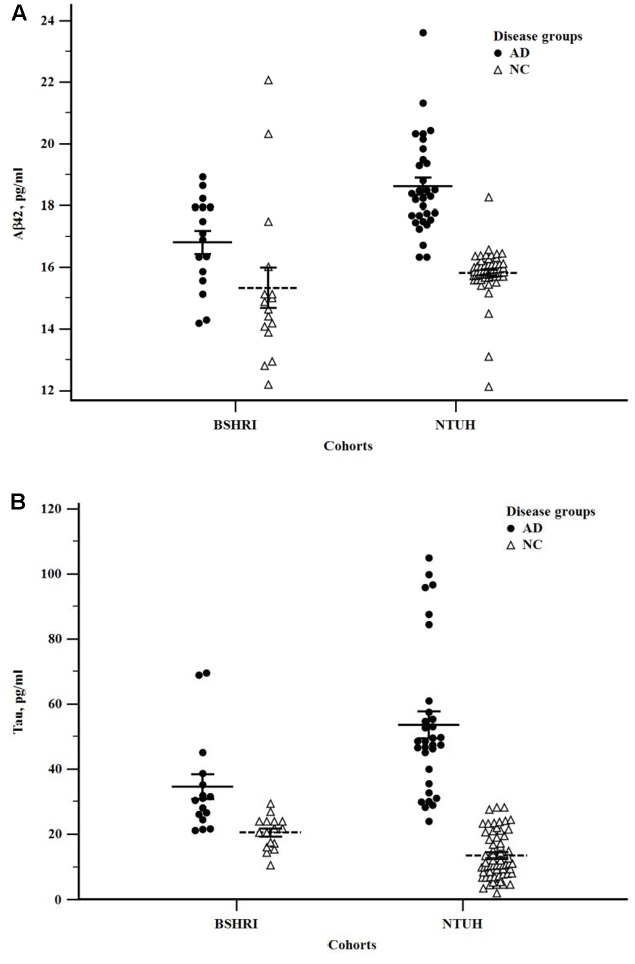
Scatter plots of Aβ42 **(A)** and tau **(B)** levels in the Banner Sun Health Research Institute (BSHRI) and National Taiwan University Hospital (NTUH) cohorts by disease groups.

We assessed if the levels of Aβ42 and tau in plasma showed disease-associated differences in the individual cohorts, and when the data were combined. Because of the presence of an age-effect, ANOCOVA with age as a covariate was used when examining the group analyte differences between ADs and NCs for the NTUH and combined cohorts. The compiled data are shown in **Table 2**. To illustrate the distribution patterns of the single analyte data, scatter plots of Aβ42 and tau levels from individual cohorts are shown in **Figure 2**.

**Table 2:** Group differences in Aβ42 and tau and their computed ratios and products.

BSHRI	NC^#^ (*N* = 16)	AD^#^ (*N* = 16)	Group differences (95% CI)^&^	*P*-value^+^
Aβ42	15.33 ± 0.66	16.8 ± 0.37	1.48 (3.0, 0.1)	0.063
Tau	20.48 ± 1.24	34.52 ± 3.75	14.0 (22.3, 5.7)	0.002
Aβ42/tau	0.79 ± 0.07	0.55 ± 0.05	-0.24 (-0.40, -0.07)	0.0061
Aβ42 × tau	316.43 ± 26.59	574.52 ± 57.80	258.1 (390.4, 125.8)	0.0006

**NTUH**	**NC**^#^ **(*N* = 61)**	**AD**^#^ **(*N* = 31)**	**Group differences (95 % CI)**^&^	***P*-value^±^**

Aβ42	15.81 ± 0.14	18.63 ± 0.21	2.82 (3.32, 2.33)	3.63e-16
Tau	13.98 ± 1.89	52.47 ± 2.72	38.48 (44.98, 31.99)	7.19e-17
Aβ42/Aβ	0.26 ± 0.012	0.52 ± 0.017	0.26 (0.30, 0.21)	1.67e-17
Aβ42/tau	1.58 ± 0.11	0.43 ± 0.16	-1.14 (-0.77, -1.52)	1.54e-07
Aβ42 × tau	222.28 ± 33.12	976.77 ± 47.77	754.49 (868.42, 640.56)	6.18e-19

**Combined**	**NC**^#^ **(*N* = 77)**	**AD^#^ (*N* = 47)**	**Group differences (95 % CI)**^&^	***P*-value**^±^

Aβ42	15.72 ± 0.16	18.00 ± 0.25	2.28 (1.68, 2.87)	3.632e-13
Tau	14.89 ± 0.81	47.09 ± 3.25	32.20 (25.46, 38.93)	<2e-16
Aβ42/Aβ	0.27 ± 0.0048	0.46 ± 0.021	0.19 (0.15, 0.24)	<2e-16
Aβ42//tau	1.42 ± 0.11	0.46 ± 0.026	-0.97 (-1.19, -0.74)	2.11e-10
Aβ42 × tau	235.50 ± 13.59	850.21 ± 59.04	614.71 (493.07, 736.35)	<2e-16

*^#^Means and standard errors. ^&^Group differences reflect the changes comparing to NC; when values in AD group are higher than NC group, the differences are negative; the lower and upper limits at 95% confidence intervals (CI) are listed within parenthesis. ^+^*P*-values from independent two-sample *t*-test. ^±^P-values from ANCOVA to examine group differences with age as a covariate*.

In both cohorts, the levels of plasma tau were significantly higher in ADs than in NCs (BSHRI: NCs: 20.48 + 1.24 pg/ml; ADs: 34.52 + 3.75 pg/ml, *P* = 0.002; NTUH: NCs: 13.98 + 1.89 pg/ml; ADs: 52.47 + 2.72 pg/ml, *P* = 7.19 × 10^-17^). There was a small but significant increase in Abβ2 levels in the AD group of the NTUH cohort (NCs: 15.81 + 0.14 pg/ml; ADs: 18.63 + 0.21 pg/ml, *P* = 3.63 × 10^-16^). This measure was not significantly different in the BSHRI cohort (*P* = 0.063) as Aβ42 levels in the NC group had significant overlap with the values from the AD group.

In the combined cohort, statistical significance for group differences were detected in tau levels: ADs had significantly higher tau levels (NCs: 14.89 + 0.81; ADs: 47.09 + 3.25, *P* = 2.0 × 10^-16^) (**Table 2**). Although group differences in Aβ42 levels were small (NCs: 15.72 + 0.16 pg/ml; ADs: 18.00 + 0.25 pg/ml, *P* = 3.6 × 10^-16^), they did reach the level of significance. In addition to single analyte differences, ratio (Aβ42/tau) and product (Aβ42 × tau) were also computed for group comparison. All cohorts showed significant differences between ADs and NCs (**Table 2**). The ratios of Aβ42 to tau were lower in ADs, while the products of Aβ42 and tau were higher in ADs (**Table 2**). The product of Aβ42 and tau was significantly increased in ADs.

### Receiver Operating Characteristic Curve (ROC) Analysis

To further evaluate the utility of individual analytes, their ratios or products in identifying probable AD, age was incorporated into a model along with biochemical measures for ROC analysis with LOO cross-validation. As shown in **Table 3**, single analytes in the BSHRI group for tau achieved 81% accuracy in identifying ADs in the BSHRI cohort, which was better than for single Aβ42 levels (69%). The accuracies for the ratio and product were 72 and 84%, respectively. With respect to the NTUH cohort, tau, Aβ42, ratio, and product all had high accuracy of identifying probable ADs.

**Table 3:** Leave-one-out receiver operation characteristics analyses of BSHRI and NTUH data.

BSHRI	Sensitivity (95% CI)^#^	Specificity (95% CI)^#^	+LR	-LR	Accuracy (95% CI)^#^	Age-dependent?
Aβ42	0.56 (0.31, 0.81)	0.81 (0.63, 1.00)	3	0.54	0.69 (0.53, 0.84)	Yes
Tau	0.75 (0.56, 0.94)	0.88 (0.69, 1.00)	6.00	0.29	0.81 (0.66, 0.94)	No
Aβ42/tau	0.63 (0.38, 0.88)	0.81 (0.63, 1.00)	3.33	0.47	0.72 (0.56, 0.88)	No
Aβ42 × tau	0.88 (0.69, 1.00)	0.81 (0.62, 1.00)	4.67	0.15	0.84 (0.72, 0.97)	No
**NTUH**
Aβ42	0.94 (0.84, 1.00)	0.97 (0.92, 1.00)	28.53	0.067	0.96 (0.91, 0.99)	Yes
Tau	0.97 (0.90, 1.00)	0.95 (0.89, 1.00)	19.68	0.034	0.96 (0.91, 0.99)	No
Aβ42/tau	0.94 (0.84, 1.00)	0.97 (0.92, 1.00)	28.53	0.067	0.96 (0.91, 0.99)	No
Aβ42 × tau	1.00 (1.00, 1.00)	0.92 (0.84, 0.98)	12.20	0.00	0.95 (0.89, 0.99)	No

*^#^95% CI: Lower, Upper. Abbreviations: CI, confidence interval; +LR, positive likelihood ratio; -LR, negative likelihood ratio*.

To explore the feasibility of establishing general criteria for identifying clinical AD across populations and age, we tested a prediction model in the combined cohort, which had subjects covering a broader age distribution and disease severity. **Table 4** summarizes the results from the combined cohorts, including sensitivity, specificity, and positive and negative likelihood ratios (LR). The cut-off values were also included in the table. The only measure that had an age-dependent cut-off value was Aβ42. Both single analytes performed well in terms of sensitivity, specificity, AUC, and accuracy. The model of Aβ42-to-tau ratio with age gave 83% sensitivity and 94% specificity. The product Aβ42 × tau with age had highest AUC (0.98, *P* = 2.55 × 10^-19^), along with 94% sensitivity, 92% specificity, 92% accuracy, +LR value at 9.22, and -LR value at 0.047.

**Table 4:** Leave-one-out (LOO) receiver operation characteristics analyses of the pooled data.

Combined cohort	Sensitivity (95% CI)^#^	Specificity (95% CI)^#^	AUC (*P*-value)	+LR	-LR	Accuracy (95% CI)^#^	Age-dependent^∗^	Cut-off value
Aβ42	0.89 (0.79, 0.98)	0.90 (0.82, 0.96)	0.92 (2.90e-15)	8.60	0.12	0.90 (0.84, 0.94)	Yes	^∗∗^
Tau	0.89 (0.79, 0.98)	0.94 (0.88, 0.99)	0.97 (8.83e-19)	13.76	0.11	0.92 (0.87, 0.96)	No	>25.41
Aβ42/tau	0.83 (0.72, 0.94)	0.94 (0.88, 0.99)	0.95 (2.11e-17)	12.78	0.18	0.90 (0.84, 0.94)	No	>0.61
Aβ42 × tau	0.96 (0.89, 1.00)	0.90 (0.83, 0.96)	0.98 (3.07e-19)	9.22	0.047	0.92 (0.87, 0.97)	No	>382.68

*^#^95% CI: Lower, Upper. Abbreviations: AUC, area under curve; CI, confidence interval; +LR, positive likelihood ratio; -LR, negative likelihood ratio. ^∗^, The cut-off value was obtained without the LOO procedure. ^∗∗^, The cut-off value was a function of age*.

## Discussion

In this study, we showed that IMR technology provided a high-sensitivity assay platform for reliably measuring plasma Aβ42 and tau in blood allowing for discrimination between NCs and clinically diagnosed ADs. This was demonstrated in two independently recruited cohorts in this study. In both cohorts, data confirmed that plasma tau levels from IMR assays had high degrees of sensitivity and specificity, which was enhanced by including computed ratios and products, Aβ42, along with age to distinguish ADs from NCs. With high-sensitivity and specificity not previously seen by other assay platforms, we developed regression models that showed the power of discrimination these measures provide. In separate cohorts, the regression model incorporating age and tau level had 81 and 96% accuracy in identifying probable AD in the BSHRI and NTUH, respectively; incorporating age with the products of Aβ42 and tau had 84% accuracy in the BSHRI cohort and 95% accuracy in the NTUH cohorts. When the two cohorts were combined, at a 382.68 (pg/ml)^2^ cut-off value, the products of Aβ42 and tau achieved 92% accuracy with 96% sensitivity and 90% specificity.

Our results strongly support plasma Aβ42 and tau levels as having a significant role in AD biomarker development. Previously, there have been few studies of plasma levels of tau in AD (Sparks et al., 2012; Zetterberg et al., 2013; Krishnan and Rani, 2014). The results from these studies showed technical limitations in measurements of tau levels in plasma. At present, only the new technologies of SIMOA and IMR have been able to produce reliable plasma tau measurements (Zetterberg et al., 2013; Mattsson et al., 2016). In the recent study by Mattsson et al. using SIMOA to measure tau, the results showed mean values at 2.58 pg/ml in NCs and 3.12 pg/ml in ADs in the Alzheimer’s Disease Neuroimaging Initiative (ADNI) cohort, and 5.58 pg/ml in NCs and 5.37 pg/ml in ADs in the Swedish Biomarkers for Identifying Neurodegenerative Disorders Early and Reliably (BioFINDER) study cohort. These values were all much lower than the values obtained with the IMR-based tau assay in this and previous studies (Chiu et al., 2013, 2014; Zetterberg et al., 2013). There were significant increases in plasma tau levels in clinical ADs compared with NCs and MCI in the ADNI cohort, but this result was not detected in the BioFINDER cohort (Mattsson et al., 2016). The distribution of plasma tau levels very much overlapped between the diagnostic groups. By contrast, the IMR assayed tau values overlapped less between diagnostic groups; AD tau levels in the BSHRI cohort were significant with 1.7-fold increased from NCs and 4-fold in the NTUH cohort; in both IMR studied cohorts, the IMR tau data was less compressed. The tau levels in ADs in the combined cohort had median values of 45.12 pg/ml in a range of 21.16–105.03 pg/ml.

This study established that in a group of patients with clinically diagnosed moderate AD, plasma samples contained elevated tau compared to NCs, whereas the levels of Aβ42 in the AD groups had either a small increase (in NTUH cohort) or an increasing trend (BSHRI cohort). However, the prediction accuracy for ADs was enhanced by the ratio or product of plasma levels of Aβ42 and tau. The improvement was more pronounced in the United States (BSHRI) cohort, increasing from 75 to 81% when using Aβ42 or tau levels alone to 88% when using Aβ42 × tau. A smaller improvement was shown in the NTUH cohorts as its single analyte measures already had high prediction accuracy (95–97%). When a logistic regression model was tested in the pooled data, which spanned a broader age range and disease severity, the prediction accuracy for clinically diagnosed AD were 93% for Aβ42 × tau.

As the NTUH cohort was not age-matched between the disease groups, age was included as a covariate for analysis of disease-group differences using ANCOVA. While significantly higher plasma Aβ42 was detected in probable ADs than in NCs in the NTUH cohort, there was only a non-significant trend in the BSHRI cohort. The lack of significance could be contributed by greater variability of Aβ42 values in NCs, smaller number of participants, and data overlap between NC and AD groups.

The most common issues for measurement of small amount of molecules in the protein-rich media such as plasma and serum are interference and matrix effects that might result in false high or low detection of analyte concentrations (Blennow and Zetterberg, 2015a). It is possible that the interactions of antibody-conjugated magnetic nanobeads and target analyte during the assay are less prone to be masked by the abundant proteins present in plasma, as magnetic nanobeads are oscillating rapidly in a magnetic field under alternate currents. Moreover, the IMR procedure does not include secondary antibody, which can be potential source of immunoglobulin cross-reactivity between species.

Plasma or serum Aβ species and tau levels measured by conventional ELISA assays have been inconsistent in demonstrating disease-associated differences due to assay sensitivity and the wide data overlap between groups (Sparks et al., 2012; Chiu et al., 2013, 2014; Zetterberg et al., 2013; Krishnan and Rani, 2014; Tzen et al., 2014) The application of IMR and SQUID technology for AD blood biomarker development has been a recent invention (Yang et al., 2011; Chiu et al., 2012, 2013, 2014). The current study is the first side-by-side comparison of IMR-assayed findings of cohorts from two distinct ethnic populations.

Plasma Aβ42 levels have been investigated extensively in autosomal dominant AD and down syndrome, as well as late-onset AD, using single or multiplex ELISA methods, where elevated plasma Aβ42 levels and/or Aβ42/40 ratios in patients with autosomal dominant AD have been reported (Cavani et al., 2000; Schupf et al., 2001; Coppus et al., 2012; Toledo et al., 2013; Fleisher et al., 2015; Quiroz et al., 2015). However, studies of sporadic AD subjects were not consistent, reporting Aβ42 levels as being unchanged, decreased, or increased (Mehta et al., 2000; Blennow et al., 2009; Lui et al., 2010; Chiu et al., 2012; Wu et al., 2012; Toledo et al., 2013; Janelidze et al., 2016; Lovheim et al., 2016; Poljak et al., 2016). Factors that affect Aβ42 results include platform-associated limitations such as matrix effect, interference, and epitope masking (Bibl et al., 2004; Toledo et al., 2011; Slemmon et al., 2012, 2015; Blennow and Zetterberg, 2015b). Although both ELISA assays and IMR assays are based on antigen-antibody reactions, IMR assays appears to have unique capability of detecting modest alterations in plasma Aβ42 levels possibly due to minimal matrix effect. However, the possibility of different forms or sub-types of Aβ42 being detected by these assay platforms cannot be ruled out. It is crucial to confirm plasma Aβ42 increases in AD in studies with larger subject numbers, since lower CSF Aβ42 levels have been shown to be very sensitive marker for pathologically confirmed AD (Shaw et al., 2009). Disease heterogeneity could be a common problem contributing to lack of differences between groups in plasma samples (Toledo et al., 2011). This is inevitable in a complex multi-factorial disease such as AD, where factors of age, gender, ApoE genotype, and other pathologies can affect disease progression. The risk genotype, ApoE *e*4, has been reported to associate with lower Aβ42 levels in plasma samples (Sun et al., 2009; Metti et al., 2013). In this study, we could not establish such relationship possibly due to too small number of subjects in BSHRI cohort, as ApoE genotype data were not available from NTUH cohort. Future study will ensure such data available for assessing the effects of ApoE genotypes on plasma Aβ and tau levels assayed by IMR.

The ability for plasma levels of Aβ42 and tau to aid in clinical diagnosis of AD were evaluated vigorously as performance metrics in this study, based on areas under the curve (AUC) of ROC, which were compared using LOO cross-validation to avoid over-fitting. The use of the logistic model approach with age took into consideration that the cut-off value of a given plasma analyte might be age-dependent. The cut-off value will not be a single value for all ages, but a series of cut-off values corresponding to different ages, when the age-effect in the logistic model is significant.

Various combinations of Aβ42, tau, or ptau levels in CSF such as ratios of tau or ptau to Aβ42 performed better for diagnosis of AD than single biomarker. This should be expected based on the known interaction of these molecules in AD. In AD CSF, Aβ42 levels were decreased and tau or ptau levels were increased (Hampel et al., 2004; Diniz et al., 2008; Mattsson et al., 2013; Toledo et al., 2013; Kang et al., 2015). Plasma Aβ42 was present in a narrow range at low concentrations with small changes between subjects. Therefore, the product with tau levels improved the usefulness of plasma Aβ42. This was supported by AUC analyses of predicting accuracy for clinically diagnosed ADs. A recent review of CSF biomarker panels indicated that regression models incorporating various combinations of factors such as age, gender, baseline MMSE, or ApoE status could increase similar robustness for the AD diagnosis (Duits et al., 2014).

There are limitations of this study that should be considered; the BSHRI cohort was small; both study cohorts were limited to clinically assessed subjects; and the study was cross-sectional. Nonetheless, the results are promising due to the high degree of discrimination between ADs and NCs, supporting that additional studies are warranted to confirm these findings. Such discrimination has not been reported for an AD core blood biomarker previously. Further work is needed (1) to clarify and confirm the assay ability to identify subjects at preclinical stages of AD, e.g., MCI; (2) to confirm discrimination AD from other types of dementia; (3) to track AD in longitudinal studies; (4) to determine correlation with other biomarkers (e.g., imaging and CSF markers); and (5) to assess the benefits of disease-modifying treatments.

## Conclusion

The results from this side-by-side study supported recent findings using the IMR platform, and extend the findings to a Caucasian population. Using ultra-sensitive IMR-based assays, patients with the clinical diagnosis of late-onset AD dementia could be identified with a high accuracy rate by combining use of Aβ42 and tau levels. Further studies are needed in larger study cohorts for differential diagnosis, early detection, and tracking of AD.

## Author Contributions

All authors listed had substantial contribution to the work described in this manuscript. The manuscript was drafted and revised to the final form by L-FL. DW provided thoughtful editing to the manuscript at various stages. The experimental design was conducted in a joint effort from L-FL, ER, MS, and M-JC. Clinical diagnosis of the subjects was performed by MS (BSHRI site) and M-JC and T-FC (NTUH site). Neuropsychological assessment was performed by CB at BSHRI site. L-FL, DW, and CS were responsible for data acquisition. Assays blinded for subject identifiers and disease groups were under the supervision of S-YY and C-CY. AG participated in data organization and assay verification. Statistical analyses were performed by NJ and NS under the supervision of KC. Data Interpretation was a joint effort from L-FL, KC, ER, MS, and M-JC.

## Conflict of Interest Statement

L-FL has received a research funding for a separate project from the MagQu Company. The MagQu Company participants only had role in performing sample assays in this work and had no role in data analysis or interpretation or decision to submit this manuscript for publication. DW, CS, NS, AG, CB, T-FC, KC, M-JC, and ER report no conflict of interest. MS report having contracts or grants with Avid, Functional Neuromodulation, Roche, Merck, vTv Therapeutics, AstraZeneca, Lilly, Lundbeck, and Biogen; serving as a consultant for Lilly, Biogen, Forum, Axovant, FujiRebio, vTv Therapeutics; and being a shareholder for Versanum and Muses Labs. MagQu Company provided assay services without charge. The other authors declare that the research was conducted in the absence of any commercial or financial relationships that could be construed as a potential conflict of interest.

## Footnotes

1 www.magqu.com

2 http://www.R-project.org/
